# Distal Anastomotic New Entries After Type A Aortic Dissection Repair: An Underreported and Underappreciated Risk?

**DOI:** 10.1016/j.atssr.2025.12.016

**Published:** 2026-01-12

**Authors:** Stefan van Dinter, Nabil Saouti, Foeke Nauta, Guillaume Geuzebroek, Tychon Geeraedts, Jan Jaap Janssen, Robin Heijmen

**Affiliations:** 1 Department of Cardiothoracic Surgery, Radboud University Medical Center, Nijmegen, The Netherlands; 2 Department of Radiology, Radboud University Medical Center, Nijmegen, The Netherlands

## Abstract

**Background:**

Acute Stanford type A aortic dissection necessitates emergency surgery. Despite proximal repair, dilation of the downstream aorta is often observed. Distal anastomotic new entry (DANE) may drive adverse remodeling and reintervention. We evaluated the incidence, recognition, and impact of DANE after hemiarch and zone 2 arch replacement.

**Methods:**

We retrospectively analyzed 42 patients with residual dissection after type A dissection surgery between January 2022 and 2024. Twenty-three underwent hemiarch and 22 zone 2 arch replacement. All had preoperative computed tomography and up to 1-year follow-up (or until reintervention) of the descending thoracic aorta. DANE was defined as contrast opacification of the false lumen contiguous with the prosthetic lumen. Outcomes included distal aortic growth (≥5 or ≥10 mm/y), maximum diameter ≥50 mm, and reintervention within 6 and 12 months.

**Results:**

DANE was identified in 19 of 42 patients (45%), with similar incidence after hemiarch (45%) and zone 2 (46%) replacement. None were reported in radiology assessments; the multidisciplinary aortic team documented 3 (16%). At 1 year, patients with DANE had larger distal aortic maximum diameters (49.2 vs 40.6 mm; *P* < .01) and were at a higher risk of ≥5 mm of growth or reintervention within 6 months (odds ratio, 48.0 [7.47-966.0]). Ninety-five percent with DANE experienced ≥5 mm of growth or reintervention within 6 months vs 26% without DANE (*P* < .01).

**Conclusions:**

DANE occurred frequently after acute dissection repair and was underreported. Its presence was associated with early distal aortic growth and reinterventions. Systematic imaging review and preventive measures against DANE may improve long-term outcomes.

**In Short**

▪ Distal anastomotic new entry (DANE) occurred in nearly half of type A dissection patients after hemiarch or zone 2 arch replacement and was strongly associated with early distal aortic growth and reintervention.
▪ DANE was largely underreported in standard radiology and multidisciplinary evaluations, underscoring the need for systematic 2-plane computed tomography assessment of the distal anastomosis.
▪ Early identification and potential endovascular sealing of DANE may prevent adverse distal remodeling and improve long-term outcomes.

Acute Stanford type A aortic dissection (ATAAD) is a life-threatening condition requiring emergent surgery. Despite successful proximal repair, residual downstream dissection and false lumen patency remain major concerns, leading to distal aortic aneurysm formation in almost 50% of patients and increasing reintervention and mortality rates.^1^ Complete false lumen exclusion is therefore crucial. The frozen elephant trunk technique has been proposed to improve remodeling but adds complexity and risk of spinal cord injury, with reintervention rates still reaching 15% after 5 years.^2^

Distal anastomotic new entry (DANE) has recently been identified as a key risk factor for adverse distal remodeling and reintervention.^3^ To combine the benefits of frozen elephant trunk while avoiding its risks, we adopted zone 2 arch replacement (Z2AR) with reimplantation of the brachiocephalic trunk and left common carotid artery, creating a proximal landing zone for endovascular completion.^4^ We hypothesized that the circular end-to-end anastomosis in Z2AR might reduce DANE compared with the more variable configuration in hemiarch replacement (HAR).

This study aimed to determine the incidence, recognition, and clinical impact of DANE after ATAAD repair with HAR or Z2AR and to evaluate its association with early distal aortic growth and reintervention.

## Patients and Methods

All patients undergoing ATAAD repair at our institution between January 2022 and 2024 were screened. Of 68 patients who were operated on, 45 had residual downstream dissection (DeBakey type 1). Of these, 23 underwent HAR and 22 Z2AR. After exclusion of 3 patients (early death or follow-up <1 year), 42 remained for analysis.

Surgery was performed under moderate hypothermic circulatory arrest (25 °C) with bilateral antegrade cerebral perfusion. Anastomoses were reinforced with external and interlaminar felt (“neomedia” technique). All procedures were performed by experienced aortic surgeons.

Computed tomography (CT) angiography imaging was evaluated preoperatively, early postoperatively, and at 1-year follow-up (or before reintervention). The descending thoracic aorta (DTA) was defined from the left subclavian artery to the diaphragm. Reintervention was indicated at >40 mm (endovascular) or >55 mm (open). Maximum diameters were measured double obliquely. DANE was defined as contrast opacification of the false lumen contiguous with the prosthetic lumen at the distal anastomosis. Two-plane alignment of the distal anastomosis ensured optimal visualization. Measurements were performed by the first author and verified by 2 interventional radiologists. Reporting of DANE was checked in radiology and multidisciplinary aortic team records.

Data were analyzed in R (R Foundation for Statistical Computing), with Student *t*-test for continuous variables and *χ*^2^ or Fisher exact test for categorical variables. Outcomes included DTA growth ≥5 or ≥10 mm/y, total diameter ≥50 mm, and reintervention within 6 or 12 months. A *P* value <.05 was considered significant.

## Results

All 42 patients had postoperative and 1-year CT follow-up (median, 396 days; Table). Twelve (29%) required a distal aortic reintervention within 1 year (median, 157 days). Overall, DANE occurred in 45% of patients, with similar incidence between HAR (45%) and Z2AR (46%).

**Table:** Demographic Data of the Total Population and Hemiarch and Zone 2 Arch Replacement Groups

Demographic Data	Total (N = 42)	HAR (n = 20)	Z2AR (n = 22)	*P*
Patient characteristics				
Sex, male	27 (64.3)	14 (70.0)	13 (59.1)	.68
Age, y	62.1	63.2	61.1	.48
Length, cm	175.7	173.8	177.4	.26
BMI, kg/m^2^	25.6	26.0	25.2	.55
GFR, mL/min	65.6	68.7	63.1	.26
LVEF				1
>50%	40 (95.2)	19 (95.0)	21 (95.5)	
31%-50%	2 (4.8)	1 (5.0)	1 (4.5)	
Comorbidities				
Diabetes mellitus	1 (2.4)	1 (5.0)	0	.48
Atrial fibrillation	2 (4.8)	1 (5.0)	1 (4.5)	1
Hypertension	17 (40.5)	9 (45.0)	8 (36.4)	.80
Hypercholesterolemia	5 (11.9)	4 (20.0)	1 (4.5)	.17
Prior myocardial infarction	2 (4.8)	1 (5.0)	1 (4.5)	1
Prior TIA or CVA	3 (7.2)	1 (5.0)	2 (9.1)	1
Prior smoker	22 (52.4)	10 (50.0)	12 (54.5)	1
Prior cardiac surgery	0	0	0	…
Connective tissue disorder	6 (14.3)	2 (10.0)	4 (18.2)	.66
Bicuspid aortic valve	3 (7.1)	3 (15.0)	0	.11
Surgical data				
Bentall (biological/mechanical)	19 (45.2)	10 (50.0)	9 (40.1)	.78
Concomitant surgery				.44
CABG	4 (9.5)	2 (10.0)	2 (9.1)	
Extrapleural LSA bypass	1 (2.4)	0	1 (4.5)	
Second DTA surgery <1 y (endovascular or open repair)	12 (28.6)	3 (15.0)	9 (40.1)	.13
DANE present postoperatively	19 (45.2)	9 (45.0)	10 (45.5)	1

Categorical variables are presented as number (percentage). Continuous variables are presented as mean.

BMI, body mass index; CABG, coronary artery bypass grafting; CVA, cerebrovascular accident; DANE, distal anastomotic new entry; DTA, descending thoracic aorta; GFR, glomerular filtration rate; HAR, hemiarch replacement; LVEF, left ventricular ejection fraction; LSA, left subclavian artery; TIA, transient ischemic attack; Z2AR, zone 2 arch replacement.

**Figure 1:**
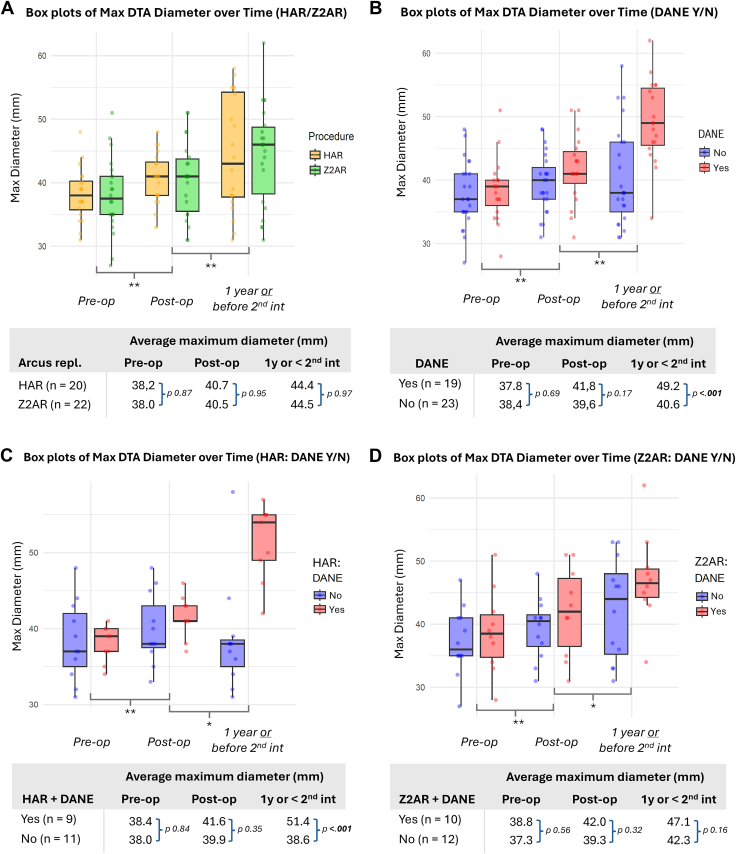
Maximum descending thoracic aorta (DTA) diameters at various perioperative moments. Boxplots show medians, interquartile ranges, and 95% CIs of maximum DTA diameters measured preoperatively, directly postoperatively, and 1 year postoperatively or before DTA reintervention within 1 year. Tables list the mean maximum diameters per time point. (A, B) Differences between hemiarch replacement (HAR) and zone 2 arch replacement (Z2AR) and between distal anastomotic new entry (DANE) and non-DANE. (C, D) Differences between DANE and non-DANE within HAR and Z2AR. ∗*P* < .05; ∗∗*P* < .001. (int, intervention; Max, maximum; Pre-op, preoperative; Post-op, postoperative; repl, replacement; Y/N, yes/no.)

Baseline and perioperative characteristics were comparable between groups. Preoperative and immediate postoperative DTA diameters were similar across repair types, yet both showed gradual growth over time (Figure 1A). At 1-year follow-up, patients with DANE exhibited significantly larger DTA diameters than those without (mean, 49.2 vs 40.6 mm; *P* < .001; Figure 1B). In patients without DANE, no relevant diameter change was observed (39.6 vs 40.6 mm; *P* = .42). Within the Z2AR subgroup, DANE was associated with marked DTA enlargement (38.6 vs 51.4 mm; *P* < .001), whereas HAR patients with DANE showed a nonsignificant increase (42.3 vs 47.1 mm; *P* = .16; Figures 1C, 1D). Demographic data did not differ between the DANE and non-DANE groups (Supplemental Table).

**Figure 2:**
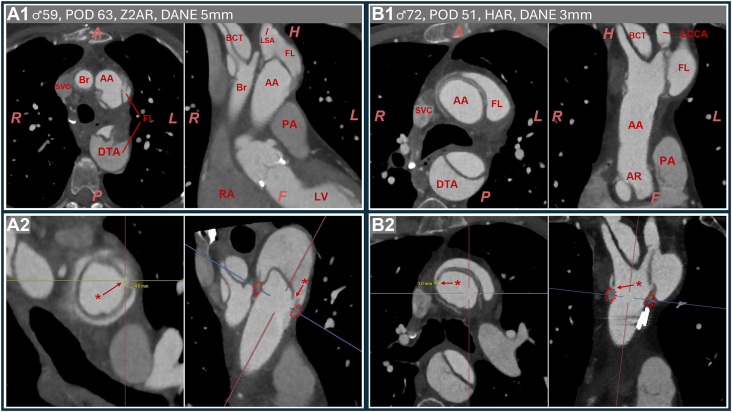
Examples of distal anastomotic new entry (DANE) identified by 2-plane distal anastomosis alignment. (A) A 59-year-old patient, 63 days after zone 2 replacement (Z2AR). (B) A 72-year-old patient, 51 days after hemiarch replacement (HAR). The top panels (A1, B1) show transversal and coronal slices of the distal anastomosis without alignment, the bottom panels (A2, B2) with 2-plane alignment revealing DANE (asterisk). Dashed red circles outline the external felt reinforcement used as alignment landmarks. (A: anterior side; AA, ascending aorta; AR, aortic root; BCT, brachiocephalic trunk; Br, prosthesis branch; DTA, descending thoracic aorta; F, foot side; FL, false lumen; H, head side; L, left side; LCCA, left common carotid artery; LV, left ventricle; LSA, left subclavian artery; P, posterior side; PA, pulmonary artery; POD, postoperative day; R, right side; RA, right atrium; SVC, superior vena cava.)

DANE recognition was poor, with none being mentioned in radiology reports and only 3 of 19 (16%) in the multidisciplinary aortic consultations. At 1-year follow-up, 95% of DANEs remained patent with persistent false lumen perfusion. Figure 2 illustrates 2 representative cases with undocumented DANE, clearly visualized after 2-plane alignment of the distal anastomosis.

**Figure 3:**
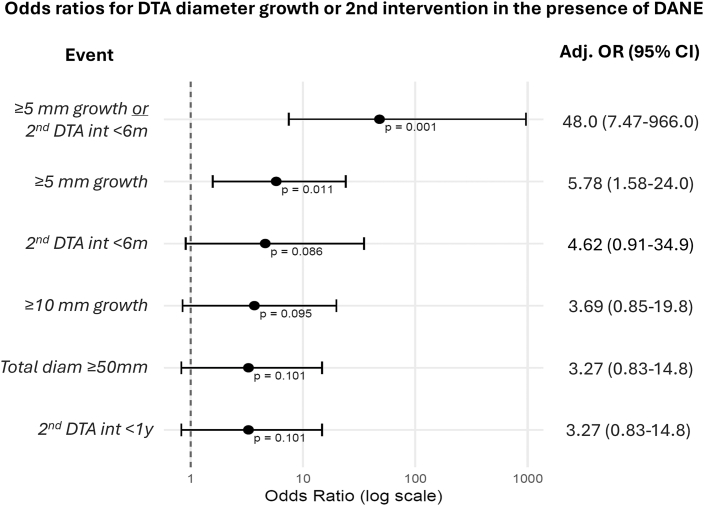
Odds ratios for descending thoracic aorta (DTA) growth or reintervention related to distal anastomotic new entry (DANE). Forest plots with adjusted odds ratios (Adj. OR) with 95% CIs for DANE-associated outcomes: DTA maximum diameter growth ≥5 mm/y and ≥10 mm/y, total diameter ≥50 mm, and early reintervention (<6 months and <1 year). (Diam: diameter; int: intervention.)

Patients with DANE had higher risk of DTA growth ≥5 mm/y (odds ratio, 5.78 [1.58-24.0]) and showed a trend toward more reinterventions within 6 or 12 months (odds ratio, 4.62 [0.91-34.9] and 3.69 [0.85-19.8]; Figure 3). In combining patients with ≥5 mm of growth or early reintervention, 95% of DANE cases met this end point vs 26% without DANE (*P* < .001).

## Comment

This study found a 45% DANE incidence after ATAAD repair, which was comparable between Z2AR and HAR and associated with early distal aortic growth or reintervention. Despite this significant clinical impact, DANE was universally underreported in clinical reports, suggesting that it is a frequently underappreciated but clinically relevant finding.

Previous studies report DANE incidences up to 70%, consistent with our findings and underscoring the fragility of a dissected aortic wall, even with felt reinforcement.^3^ Contrary to our hypothesis, Z2AR did not lower DANE occurrence, implying that anastomotic geometry alone does not affect DANE incidence.

Techniques aiming to reduce DANE formation have been explored. The Ascyrus Medical Dissection Stent (AMDS; Artivion) combines a felt-reinforced ring and bare stent to stabilize the distal anastomosis and true lumen, reportedly lowering DANE rates by 30% and improving false lumen thrombosis.^3^ Similarly, intimal relayering using intimal reinforcement with a prosthetic graft showed no anastomotic dehiscence in a small series.^5^ These concepts are promising but require larger and long-term studies to establish durability and optimal extent of reinforcement.

Clinically, DANE was linked to rapid aortic dilation and early reintervention in nearly all affected patients. This parallels previous reports associating DANE with distal growth and reintervention rates up to 45%.^6^ Nevertheless, DANE on its own was not mentioned as an indication for reintervention, which alongside the observed consistent underreporting likely reflects limited awareness of DANE’s impact. Two-plane CT evaluation of the distal anastomosis proved critical for DANE detection and should be incorporated into standardized postoperative assessment. Even small DANEs may maintain false lumen perfusion, driving adverse remodeling.

Preemptive thoracic endovascular aortic repair may benefit ATAAD patients with DANE, given its role in promoting favorable remodeling in both type A and type B dissections.^4^^,^^7^^,^^8^ Early or subacute thoracic endovascular aortic repair (<3 months) has been linked to fewer complications and better long-term survival in uncomplicated type B dissection.^9^ Although its role after ATAAD remains unclear, Z2AR offers an ideal proximal landing zone, allowing hybrid endovascular completion with subclavian-carotid bypass or single-branched endografts. This may be particularly useful for sealing DANE after Z2AR. However, further studies are needed to balance the benefits of early endovascular repair against procedural risks in this population.

This study’s limitations include the single-center design, small sample size, and possible influence of surgical technique or surgeon experience. The seemingly stronger impact of DANE after Z2AR may relate to earlier endovascular interventions being more feasible in this group, whereas HAR patients often underwent later repairs. Furthermore, distal aortic growth is multifactorial, in which other reentries, dissected arch vessels, connective tissue diseases, or hemodynamic factors may contribute.^10^ Larger multicenter studies with multivariable modeling are required to clarify independent predictors of distal remodeling and to refine risk stratification.

In conclusion, DANE occurred in nearly half of ATAAD patients after HAR or Z2AR and was strongly associated with early distal aortic growth and reintervention. Despite this, DANE remained largely unreported in standard radiology and multidisciplinary assessments. Routine 2-plane CT evaluation of the distal anastomosis is recommended to improve detection. Patients with early DANE may benefit from preemptive endovascular sealing, particularly when Z2AR provides a suitable landing zone. Future research should evaluate preventive reinforcement strategies and develop risk models integrating DANE and other factors predicting adverse distal remodeling after ATAAD repair.
