# Supplementary material for: Distal Anastomotic New Entries After Type A Aortic Dissection Repair: An Underreported and Underappreciated Risk?
Source: Ann Thorac Surg Short Rep. 2026 Jan 12;4(2):531–7. doi: 10.1016/j.atssr.2025.12.016 (PMC13245507; doi:10.1016/j.atssr.2025.12.016)
Supplement: Supplementary Table [file mmc1.docx]

## Supplementary data

### Supplementary table 1

| **Patient Demographic Data (n (%))** | | | | |
| --- | --- | --- | --- | --- |
|  | **Total (n=42)**  **n (%)** | **No (n=23)** | **DANE present**  **Yes (n=19)** | **p** |
| **Patient characteristics** |  |  |  |  |
| Gender (=male) | 27 (64.3) | 14 (60.9) | 13 (68.4) | 0.85 |
| Age | 62.1 | 62.8 | 61.3 | 0.62 |
| Length | 175.7 | 173.4 | 178.4 | 0.10 |
| BMI | 25.6 | 25.8 | 25.3 | 0.66 |
| GFR | 65.6 | 66.4 | 65.2 | 0.80 |
| LVEF  >50%  31-50% | 40 (95.2)  2 (4.8) | 21 (91.3)  2 (8.7) | 19 (100)  0 (0) | 0.49 |
| **Comorbidities** |  |  |  |  |
| DM | 1 (2.4) | 1 (4.4) | 0 | 1 |
| AF | 2 (4.8) | 1 (4.4) | 1 (5.3) | 1 |
| HT | 17 (40.5) | 10 (43.5) | 7 (36.8) | 0.90 |
| HC | 5 (11.9) | 4 (17.4) | 1 (5.3) | 0.36 |
| Prior MI | 2 (4.8) | 1 (4.4) | 1 (5.3) | 1 |
| Prior TIA/CVA | 3 (7.2) | 1 (4.4) | 2 (10.5) | 0.58 |
| Prior smoker | 22 (52.4) | 13 (56.5) | 9 (47.4) | 0.78 |
| Prior card surg | 0 | 0 | 0 | - |
| Connective tissue diss | 6 (14.3) | 3 (13.0) | 3 (15.8) | 1 |
| Bicuspid AoV | 3 (7.1) | 1 (4.4) | 2 (10.5) | 0.57 |
| **Surgical strategy** |  |  |  |  |
| Hemi-arch repl. | 20 (47.6) | 11 (47.8) | 9 (47.4) | - |
| Zone 2 arch repl. | 22 (52.4) | 12 (52.2) | 10 (52.6) | 1 |
| Bentall (bio or mech) | 19 (45.2) | 7 (30.4) | 12 (63.2) | 0.07 |
| Concomitant surgery  CABG (vein graft) Extra-pleural LSA bypass  2^nd^ DTA surgery within 1y (endovascular or open repair) | 4 (9.5)  1 (2.4)  12 (28.6) | 3 (13.0)  1 (4.4)  4 (17.4) | 1 (5.3)  0  8 (42.1) | -  0.80  0.16 |

**Supp. Table 1** – Demographic data of the included patients, showing the total population and population divided with distal anastomotic new entries (DANE) present and without DANE.

AF: atrial fibrillation; AoV: aortic valve; BMI: body mass index; CABG: coronary artery bypass grafting; CVA: cerebrovascular accident; DM: diabetes mellitus; DTA: descending thoracic aorta; HC: hypercholesterolemia; HT: hypertension; MI: myocardial infarction; LVEF: left ventricular ejection fraction; LSA: left subclavian artery; repl: replacement; TIA transient ischemic attack;
